# Efficacy of a novel combination of aloe vera gel and pineapple extracts in improving post-extraction pain and healing: A Randomized Controlled Trial

**DOI:** 10.4317/jced.61640

**Published:** 2024-07-01

**Authors:** Srishti Srivastava, Viraj-Rajeev Kharkar, Saudamini More, Harjit-Singh Kalsi, Sanpreet-Singh Sachdev

**Affiliations:** 1 BDS. Intern, Department of Oral and Maxillofacial Surgery Institute Dental College and Hospital, Navi Mumbai; 2 MDS. Associate Professor, Department of Oral and Maxillofacial Surgery Institute Dental College and Hospital, Navi Mumbai; 3 MDS. Assistant Professor, Department of Community Dentistry Institute Dental College and Hospital, Navi Mumbai; 4 MDS. Professor and Head, Department of Oral and Maxillofacial Surgery Institute Dental College and Hospital, Navi Mumbai; 5 MDS. Assistant Professor, Department of Oral Pathology and Microbiology Institute Dental College and Hospital, Navi Mumbai

## Abstract

**Background:**

Absorbable gelatin sponges are able to reduce the incidence of post-extraction complications when soaked with antimicrobial agents. However, the drawbacks associated with the injudicious use of antibiotics warrant the need to explore alternatives to the existing drugs. Aim: The present study aimed to evaluate the efficacy of an absorbable gelatin sponge soaked in a combination of aloe vera and pineapple extracts in reducing post-operative pain and improving the healing rate following dental extractions.

**Material and Methods:**

Patients aged 18 to 60 years undergoing a single dental extraction of a posterior tooth were provided with either plain absorbable gelatin sponge (control group) or absorbable gelatin sponge soaked in freshly prepared solution of aloe vera gel and pineapple extracts. The pain levels were measured after one, three, seven, and fourteen days post-extraction. The socket healing status was evaluated by Landry Turnbull and Howley Index after one and two weeks post-extraction respectively.

**Results:**

The mean pain was significantly less (*p*<0.05) for patients in the experimental group on the 1st, 3rd, and 7th postoperative days as compared to the patients in the control group. No significant differences (*p*>0.05) were observed in the healing status between the two groups at any time interval.

**Conclusions:**

The present study found the combination of aloe vera gel and pineapple extracts to be effective in reducing post-operative pain following dental extractions. While the improvement in the healing rate failed to reach statistical significance in the present study, generally less inflammation was observed in sockets treated with absorbable gelatin sponge soaked in the a combination of aloe vera and pineapple extracts.

** Key words:**Post-extraction pain, Socket healing, Aloe Vera, Bromelain.

## Introduction

The alveolar bone socket following extraction of a tooth may be considered a wound that undergoes healing over a period of three weeks. During this time, infection of the socket leads to several complications such as pain, swelling, excessive bleeding, delayed healing, and the development of dry socket (1). Absorbable gelatin sponges (AbGel) are routinely employed as hemostatic aids following dental extractions (2,3). AbGel is often soaked with antimicrobial agents to simultaneously reduce the incidence of post-operative infections. This combination has proven effective in preventing the occurrence of dry sockets following dental extractions (3,4).

Prophylactic antibiotics are also routinely prescribed following dental extractions or other oral surgical procedures. While they form an integral part of the therapeutic and preventive dental practice, certain problems related to their use cannot be overlooked. Injudicious or repetitive use of antibiotics is associated with the development of resistant bacterial strains which may in turn cause more harm than benefit to the patient (5). Thus, the prescription of antibiotics may rightly be considered a double-edged sword in dentistry owing to the sequela of antibiotic resistance. To tackle the issue, recent emphasis has shifted to the use of natural alternatives for antibiotics in controlling post-operative infection following oral surgical procedures (6).

Since ancient times, one of the most commonly used herbal ingredients with antimicrobial and anti-inflammatory properties in the field of medicine is aloe vera. Its leaves are composed mainly of water (About 98%), and the remainder of vitamins, minerals, enzymes, polysaccharides, and other water-soluble organic acids (7). The medicinal properties of aloe vera are attributable to certain immunomodulatory components such as acemannan and phytosterols (8). These components are able to actively stimulate growth factor production, fibroblast proliferation, and collagen deposition ultimately hastening the wound healing process (9). Its efficacy in improving the rate of healing along with reduction of pain and inflammation in periodontal surgeries, reduction of pocket depths when placed with coepack, bleeding gums, aphthous stomatitis, oral lichen planus, and as a lubricant in root canals has been demonstrated across various studies conducted over time (10-13).

Bromelain, a proteolytic enzyme found in the stem and fruit of pineapple possesses various therapeutic benefits that have been harnessed in the field of medicine (14). It has demonstrated its efficacy in modern medicine as a remedy for conditions such as blood disorders, bronchitis, angina pectoris, sinusitis, and digestive problems owing to its ability to control inflammatory events related to the action of cytokines (14-16). By virtue of its anti-inflammatory properties, bromelain has also been demonstrated to be efficacious in reducing post-operative complications following dental procedures such as third molar extractions and periodontal surgeries (17,18).

Although some studies have demonstrated the efficacy of aloe vera and bromelain in improving the healing rate of dental sockets and reducing the post-operative complications following dental extractions, their practical applicability warrants further exploration (17-20). None of the earlier studies have attempted to utilize a combination of the two materials for improving the post-extraction healing rate. Additionally, the use of AbGel in conjunction with the two extracts has also not yet been studied to date. In this context, the present study aimed to evaluate the efficacy of an AbGel soaked in a combination of aloe vera and pineapple extracts in reducing post-operative pain and improving the healing rate following dental extractions.

## Material and Methods

-Study Design.

The present randomized controlled trial was conducted over a duration of two months from September to November 2023. The study adhered to the principles of the Declaration of Helsinki and the protocol was approved by the Institutional Ethical Committee (Ref No: BEC41908202, Dated 09/08/2023). A total of 108 patients requiring extraction of a posterior tooth were recruited from the institutional Department of Oral and Maxillofacial Surgery (OMFS). Informed consent was obtained from the patients following which a detailed case history was elicited.

-Selection criteria:

Patients of age ranging from 18 to 60 years having a single completed erupted posterior tooth indicated for extraction due to dental caries or for orthodontic treatment. Patients with systematic disorders or conditions that could affect healing were excluded from the study. Pregnant and lactating women were also excluded. Patients with periapical lesions larger than 3 mm in dimensions, periodontitis, or ankylosis associated with the tooth to be extracted were excluded from the study. A preliminary hemogram of the patients was advised to confirm the absence of any significant blood-related abnormalities. The patients selected after the application of the inclusion and exclusion criteria were recalled the next morning for extraction. The patients were randomly assigned to the experimental group (AbGel soaked in Alopine solution) or the control group (plain AbGel) using the coin-flip method.

-Preparation of the ‘Alopine’ solution:.

On the day of extraction, one leaf was freshly cut from an aloe vera plant using a knife at its base. A 5 mm tip portion of the leaf and the peripheral spikes were removed, and the sap was allowed to drip for five minutes. The leaf was then soaked in water for 15-20 minutes to remove any remaining latex. The outer layer of the leaf was peeled to remove the gel into a glass jar.

**Figure 1:**
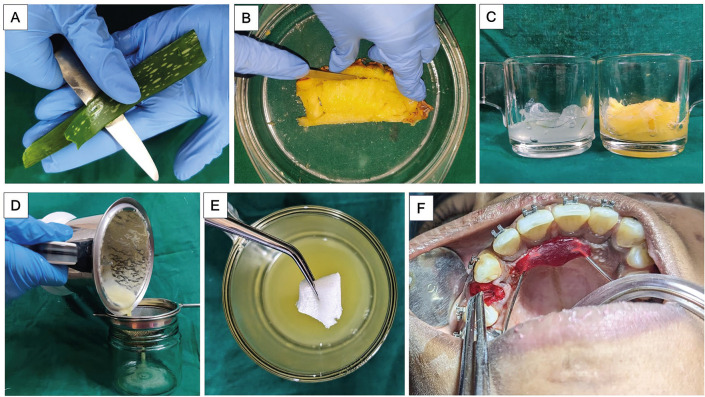
A) Extraction of aloe vera gel; B) Separation of the pulp of pineapple; C) Equal amount of aloe vera gel and pineapple extract; D) Straining of the blended extracts into a glass jar; E) Soaking the absorbable gelatin sponge in the experimental solution; F) Placement of the soaked gelatin sponge into the extraction socket.

A similar quantity of extract (1:1) was obtained from freshly cut pineapple pieces and blended with the aloe vera gel. The prepared ‘Alopine’ (aloe vera + pineapple) solution was then strained into an air-tight glass bottle and stored at 4oC in a refrigerator. The entire procedure of preparation of the experimental ‘Alopine’ solution is depicted in Figure 1.

-Surgical procedure.

The patients were instructed to have a meal one hour before the extraction procedure. All the surgical procedures were conducted with standard aseptic precautions in a sterile environment. Local anesthetia was administered using 2% lignocaine with 1:80000 adrenaline following which the tooth was extracted. Only patients in which the tooth was extracted atraumatically without requiring surgical exposure were included in the subsequent data analysis. Depending on the group, AbGel of dimensions, either plain (control group) or soaked in ‘Alopine’ solution for 20 seconds (experimental group) was inserted and adapted into the extraction socket. Both, the patient as well as the operator were blinded regarding the type of AbGel used. A cotton roll was placed on the socket and the patient was instructed to hold it with teeth occluded for one hour. Routine post-extraction instructions were given.

Patients were provided with 650 mg Paracetamol which was to be taken in case of occurrence of pain beyond tolerable limits. Patients taking the analgesic medication were excluded from the study. Patients having excessive bleeding post-extraction were excluded from the study and the bleeding was managed by electrocautery or suturing. Curettage and antimicrobial treatment were provided to patients developing post-surgical infection and dry socket during the follow-up period and these patients were also excluded from the final data analysis.

**Figure 2:**
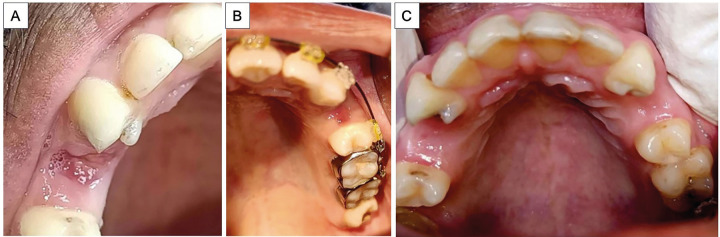
Post-operative healing in A) Control Group and B) Experimental Group after one week; C) Healing at both sites after two weeks.

**Figure 3:**
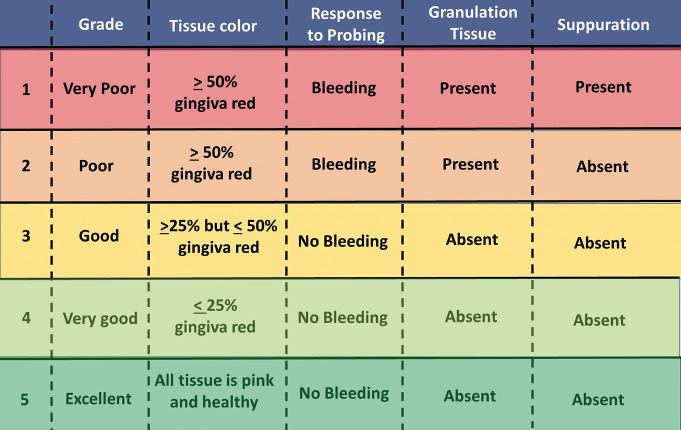
Landry Turnbull and Howley Index used for grading the socket healing status in the present study.

-Outcome assessment:

The patients were explained about the visual analog scale (VAS) and were asked to record their pain level on a scale of 0 to 10. The pain levels were recorded at 1-day, 3-day, 7-day, and 14-day post-extraction intervals by means of telephone. Evaluation of the socket healing was performed on the 7th and 14th postoperative days (Fig. 2) using the Landry Turnbull and Howley Index (Fig. 3) which involves the evaluation on a scale of 1 to 5 based on the tissue color, response to palpation, and the presence of granulation tissue or suppuration (21).

-Statistical Analysis.

The data was analysed using Microsoft Excell and analysed using IBM Statistical Package for Social Sciences Software version 20.0 (Armonk, NY, USA IBM Corp). For analysis treatment groups were considered independent variables. Pain and healing were the dependent variables. The data was found to be non-normally distributed for both groups, when assessed using Shapiro Wilk test for normality. Mann-Whitney U test was used to compare the pain and healing between two groups. The level of significance was set at *p* < 0.05.

## Results

**Figure 4:**
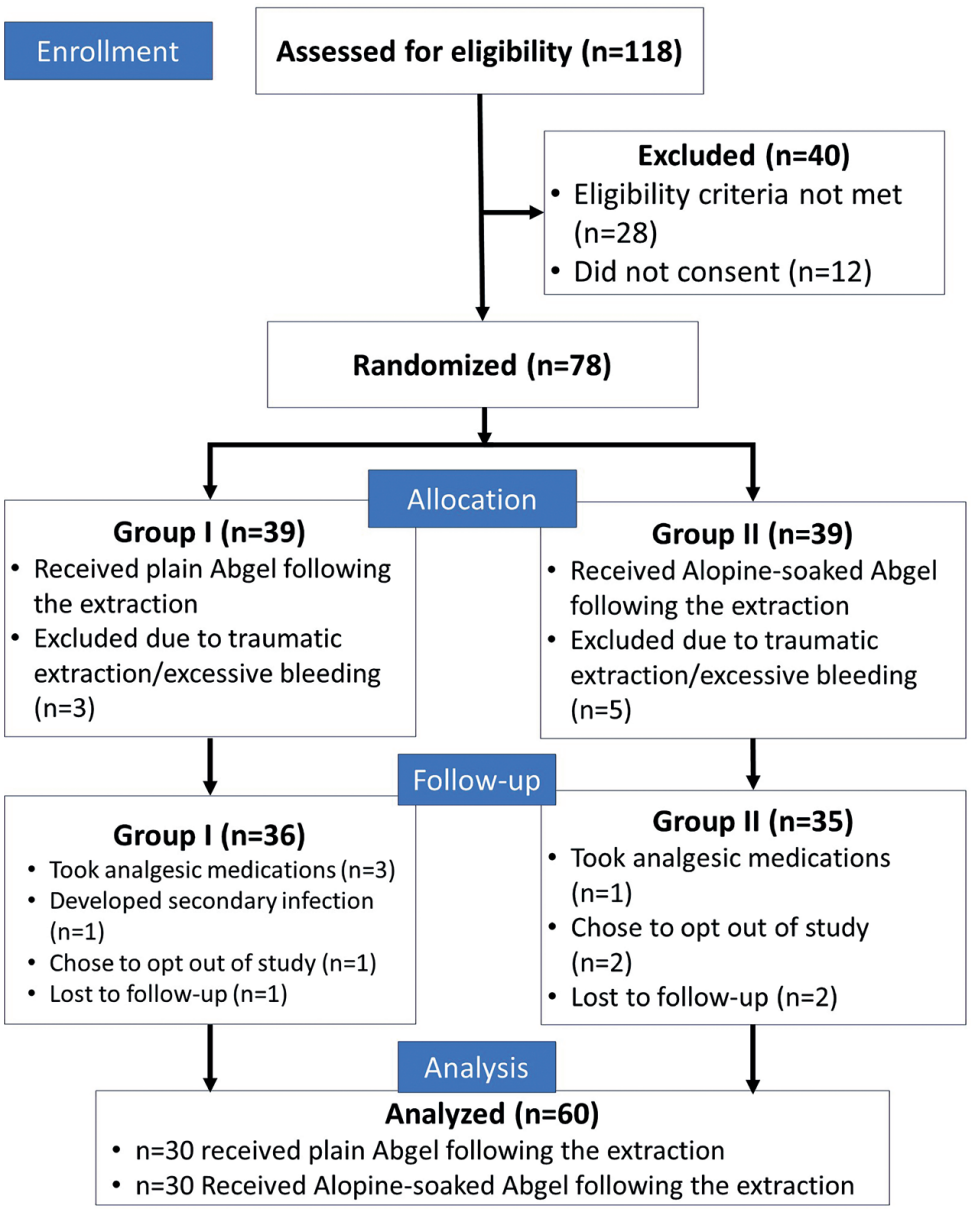
CONSORT Flow diagram indicating the selection process of the patients in the present randomized controlled trial.

A total of 60 patients were included in the final data analysis with n=30 patients in each group (Fig. 4). The study population comprised 24 males and 36 females with each group having 12 males and 18 females respectively. The mean age of the patients was 31.97 + 12.34 years in the control group and 41.39 + 10.58 in the experimental group.

There was no statistically significant difference (*p*>0.05) between the number of patients of either gender in both groups and between the mean age of the two groups. This indicated that the baseline demographic characteristics of the study population in both groups were identical and randomization performed was satisfactory.

The mean pain was significantly less (*p*<0.05) for patients in the experimental group on the 1st, 3rd, and 7th postoperative day as compared to the patients in the control group. No significant differences (*p*>0.05) were observed in the healing status between the two groups at any time interval.

## Discussion

Post-operative infections and pain are the commonest complications of dental extractions for which antibiotics constitute the mainstay of treatment and prevention (1). Keeping in mind the drawbacks associated with the injudicious use of antibiotics and analgesics, the present study aimed to determine the therapeutic efficacy of the novel ‘Alopine’ formulation in the healing of extraction sockets. The objective of the study was to develop natural alternatives to the use of antimicrobials following dental extraction which have minimal adverse effects.

Our results indicated that the post-operative pain levels until one-week post-extraction were significantly lesser in patients provided with the Alopine-soaked AbGel. No significant difference was observed in the pain levels after two weeks between the two groups. Even so, the inflammatory component in the tissues was observed to be lesser for the experimental group at all time intervals. This could be attributable to selective inhibition of the pain-promoting prostaglandins by the release of products similar to endogenous protease plasmin by Bromelain (22). Bromelain is able to regulate the release of pro-inflammatory cytokines such as prostaglandin E2, bradykinin, thromboxane A2, substance P, and plasma fibrinogen which accounts for the reduced inflammation of tissues clinically (23), ( Table 1).

Our findings corroborate with those from an earlier study conducted by de la Barrera-Núñez *et al*. that found bromelain to be effective in reducing the inflammation following the extraction of impacted third molars when administered in doses of 150 mg daily (24). Ordesi *et al*., in a similar study, found a reduced incidence of postoperative pain, erythema, and edema in patients prescribed with 50 mg bromelain daily for seven days.(25) The authors also reported that patients taking bromelain required fewer analgesics as compared to those taking paracetamol.

Aloe vera is also able to inhibit the production of various inflammatory cytokines including Tumor Necrosis Factor-α, Interleukin-6, and Interleukin-8 contributing to the reduction of inflammation-mediated pain (26). Furthermore, aloe vera causes a reduction in leukocyte adhesion ability and an increase in the anti-inflammatory Interleukin-10 levels (27). These inhibitory effects of aloe vera on inflammation and pain are owing to its phenylalanine, tryptophan, and salicylic acid components.(28) Saponins, another glycoside component present in aloe vera gel, is an antiseptic agent that cleanses the wound area and minimizes the risk of infection (29).

Although the healing rate was also improved in patients provided with AbGel soaked in the ‘Alopine’ solution, it failed to reach statistical significance. Contrasting findings were reported by Nimma *et al*. (2017) wherein significantly improved healing was achieved by aloe vera gel in patients following extraction (19). The regenerative properties of aloe vera are attributed to a constituent compound glucomannan, which is structurally similar to mannose (30). Acemannan, a polysaccharide component of aloe vera, also boosts the immunity level by stimulating the production of antibodies, facilitating clot production, and subsequently reducing the incidence of secondary infections (8).

These compounds essentially providing the beneficial effects of aloe vera are present within the gel component of the leaf held together by the mucilage layer. The gel is highly hydrophilic, able to retain large amounts of water, forming a polymeric ‘hydrogel’ (31,32). This property is particularly useful when incorporating the aloe vera gel in the moist oral environment (31). A study by Dewi *et al*. has demonstrated the ability of Aloe vera hydrogel to increase the number of fibroblasts in the bony sockets following dental extractions in rats.(20) Hence, the gel component of the leaf was utilized to promote socket healing the present study.

An added advantage of using the AbGel in combination with the mucilaginous gel was that it became easy to place and adapt the materials into the socket without harming the tissues and also achieving homeostasis at the same time. The gel being resorbable, does not require any additional procedures to be performed by the surgeon or the patient. It also eliminates the need for patient compliance which was an observed drawback in the earlier studies when the compounds were to be taken orally (24,25). Another cellulose-based substance present in the hydrogel, Liginin, confers the ability to penetrate mucosal and dermal surfaces effectively (32).

None of the patients reported the occurrence of any adverse events with the use of Alopine gel. The extracts being derived from natural plants, can be considered safe to use with almost completely free of any side effects. The only safety concerns about the use of aloe vera are in patients allergic to Lilac family and pregnant women where its use may cause abdominal cramps, diarrhoea, or constipation counteracting the electrolyte balance (33,34). In normal healthy individuals, aloe vera is largely free of any adverse effects except for slight redness or burning sensation when applied to the skin in some individuals (35). Likewise, bromelain is also not associated with any adverse effects with a handful of cases reported to date that experienced gastrointestinal adverse events (diarrhoea, nausea, and flatulence) or headache, dry mouth, and allergic reactions (36). The only drawback observed with the use of Alopine in the present study was that the extracts had to be freshly obtained and the solution freshly prepared on the same day of the procedure. The aspect of storage methods and extension of the shelf life of the solution requires further investigation.

Certain limitations of the present study need to be acknowledged. The relatively small sample size warrants further investigation for extrapolating the results to the general population. Observing the microbial count in the socket would have provided insights into the antimicrobial properties of the solution, which was not performed in the present study owing to feasibility constraints. The addition of microscopic or biochemical methods for the evaluation of the healing socket would also provide in-depth visualization of the improved healing achieved by the Alopine solution.

While the present study provided promising results, further trials with larger sample sizes and longer follow-up periods are recommended to confirm the beneficial effects of the combination of aloe vera and pineapple extracts in post-extraction socket wound healing.

## Conclusions

The present study found ‘Alopine’, a combination of aloe vera and pineapple extracts to be effective in reducing post-operative pain following dental extractions. While the improvement in the healing rate failed to reach statistical significance in the present study, generally less inflammation was observed in sockets treated with AbGel soaked in the Alopine solution. These observations point toward the potential of the novel solution in reducing inflammation and promoting healing in the socket wounds following dental extraction. Use of organic solutions would also minimize the use of anti-microbial and anti-analgesic drugs, and the harmful consequences associated with their injudicious use.

## Figures and Tables

**Table 1:** Pain levels and healing scores for both groups at various time intervals following extraction.

Post-operative interval	Control Group	Experimental Group	Sum of Ranks (W)	Z Value	p Value
Pain levels					
Day 1	4	2	1234	2.93	0.002*
Day 3	3	1	1201.5	2.99	0.001**
Day 7	2	0	1153	2.00	0.02*
Day 14	0	0	1055	1.64	1.64
Healing Score					
Day 7	4	4	959	1.64	0.23
Day 14	0	0	1055	1.64	0.08*

## Data Availability

The datasets used and/or analyzed during the current study are available from the corresponding author.
